# Effects of BNT162b2 mRNA vaccine on COVID-19 infection and hospitalisation amongst older people: matched case control study for England

**DOI:** 10.1186/s12916-021-02149-4

**Published:** 2021-10-18

**Authors:** Thomas F. D. Mason, Matt Whitston, Jack Hodgson, Ruth E. Watkinson, Yiu-Shing Lau, Omnia Abdulrazeg, Matt Sutton

**Affiliations:** 1 NHS England & NHS Improvement, Quarry House, Quarry Hill, Leeds, West Yorkshire LS2 7UE UK; 2 Health Organisation, Policy & Economics, School of Health Sciences, University of Manchester, Manchester, UK

**Keywords:** COVID-19, Vaccines, Infections, Observational study

## Abstract

**Background:**

The BNT162b2 mRNA vaccine has been shown to be effective at preventing serious COVID-19 events in clinical trials. There is less evidence on effectiveness in real-world settings, especially for older people. Here, we aimed to estimate vaccine effectiveness in the context of the rapid NHS mass-vaccination programme in England, exploiting age-based vaccination eligibility thresholds to minimise and correct for selection bias.

**Methods:**

We studied 170,226 individuals between the ages of 80 and 83 years from community settings outside care homes who received one dose of BNT162b2 mRNA between the 15 and 20 December 2020 and were scheduled a second dose 21 days later. We matched these vaccine recipients to slightly younger (aged 76–79 years) persons not yet eligible to receive the vaccine on gender, area of residence, area deprivation, health status, living arrangements, acute illness, and history of seasonal flu vaccination. We compared their rates of COVID-19 positivity and hospitalisation in the subsequent 45 days. We adjusted for the increasing concentration of COVID-19 positivity in the control population caused by the requirement to have no COVID-19 symptoms prior to vaccination.

**Results:**

Emergency hospital admissions were 51.0% (95% confidence interval 19.9 to 69.5%) lower and positive COVID-19 tests were 55.2% (40.8 to 66.8%) lower for vaccinated individuals compared to matched controls 21 to 27 days after first vaccination. Emergency admissions were 75.6% (52.8 to 87.6%) lower, and positive COVID-19 tests were 70.1% (55.1 to 80.1%) lower 35 to 41 days after first vaccination when 79% of participants had received a second dose within 26 days of their first dose.

**Conclusions:**

Receipt of the BNT162b2 mRNA vaccine is effective at reducing COVID-19 hospitalisations and infections. The nationwide vaccination of older adults in England with the BNT162b2 mRNA vaccine reduced the burden of COVID-19.

**Supplementary Information:**

The online version contains supplementary material available at 10.1186/s12916-021-02149-4.

## Background

Several vaccines against severe acute respiratory syndrome coronavirus 2 (SARS-CoV-2) infection and the resulting coronavirus disease 2019 (COVID-19) have been demonstrated to be safe and highly effective in phase 3 randomised clinical trials, with efficacy estimates for prevention of symptomatic disease ranging from 62 to 95% [1–4]. However, it is also important to examine their effectiveness when deployed in mass vaccination campaigns across diverse populations, where trial exclusion criteria do not apply and where deviations from dosing and handling protocols may occur.

Early evidence from a matched case-control study of mass vaccination using the BNT162b2 mRNA COVID-19 vaccine in Israel estimated real-world effectiveness consistent with reported trial efficacy [3, 5]. This indirectly provided evidence that vaccine effectiveness was maintained against the more transmissible B.1.1.7. (Alpha) variant [5, 6], which was widespread in the population during the study period. Vaccine effectiveness estimates were consistent across age groups, though they were slightly lower amongst people with multiple coexisting health conditions [5]. Similarly, estimates from Scotland [7] and England [8, 9] provide further early evidence of effectiveness. However, such non-randomised matching studies may be biased by systematic differences between intervention and control groups and between those receiving the intervention at different points in time. The remarkable speed of COVID-19 vaccination rollouts [5, 7–9] and specific prioritisation of vulnerable groups [10] heighten the risk of these biases, as acknowledged in existing studies [5, 7–9].

We exploit age-based eligibility phasing in the early stages of the nationwide National Health Service (NHS) population vaccination programme in England to estimate the real-world effectiveness of the BNT162b2 mRNA vaccine. We match vaccinated persons aged 80 to 83 years to slightly younger persons who did not become eligible for the vaccine until three weeks later and compare their rates of COVID-19 infection and hospitalisation over the 45 days following the date of their first dose. It is particularly important to assess real-world vaccine effectiveness in this older population group, because severe COVID-19 is strongly age-associated [11] and adaptive immune responses decline with age [12].

## Methods

### Data

MW and JH obtained population-wide person-level data for England, including vaccination details (date, type and dose), SARS-CoV-2 tests (date, result), age, gender, area of residence, use of hospital services and dates of death. For data sources, linkage methods and access, see Appendix 1. Data were extracted on 9 February 2021 and include complete records to 3 February 2021.

### Outcomes

We examined rates per 100,000 people for three outcomes: SARS-CoV-2 infection and COVID-19 related hospital attendances and hospitalisations. Infection was recorded by specimen date of SARS-CoV-2 positive polymerase chain reaction (PCR) test results from health care facilities (called Pillar 1 testing) or community testing (Pillar 2). COVID-19 related emergency department attendances were measured using diagnosis information from emergency department records combined with linked positive COVID-19 test results from 14 days before to 6 days after the attendance (see Appendix 3). COVID-19 related hospital admissions were measured using admitted patient care spell records available on discharge. The required information is available for 95% of all emergency department attendances and 93% of all emergency admissions.

### Study design

The first phase of the NHS vaccination programme in England targeted: (1) front-line health and social care workers, (2) older care home residents and their carers, and (3) people aged 80 years and over [10]. Differences in risks of exposure and outcome within the first two groups are not effectively measured in administrative datasets. We therefore focused on 171,931 individuals aged 80–83 years not living in care homes that received their first dose between 15 and 20 December 2020, of whom 78.8% received a second dose within 26 days.

**Fig. 1:**
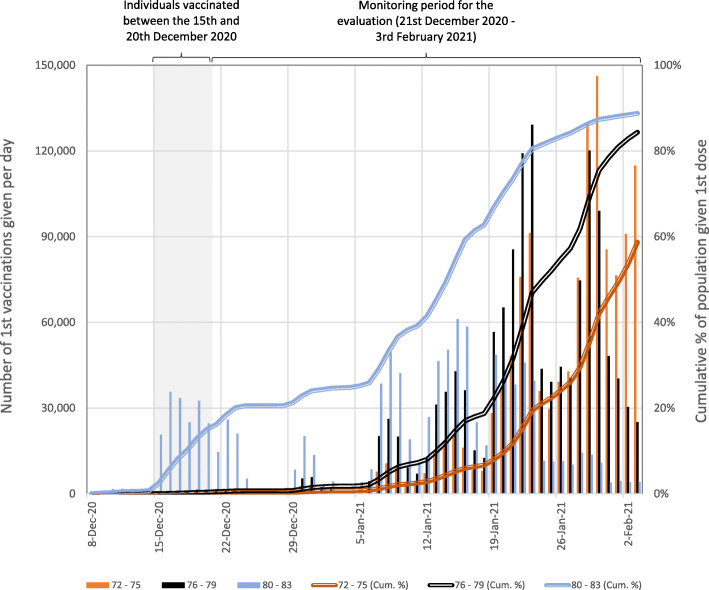
Numbers of people in England who received their first COVID-19 vaccination dose between 8 December 2020 and 3 February 2021 by age group. The cumulative totals are relative to estimates of eligible population based on extracts from the National Health Application and Infrastructure Services (NHAIS) system as of the 15 November 2020. Prior to the 4 January 2021, all individuals received the BNT162b2 mRNA vaccine, after which individuals were vaccinated with either the BNT162b2 mRNA or ChAdOx1 adenovirus vector vaccines

We compared vaccinated cases to slightly younger people aged 72–79 years who became eligible for vaccination later. The speed of vaccination rollout meant many of these received a first vaccine dose during the follow-up period (see Fig. 1). We identified suitable controls for each day of the follow-up period as those who had not received their first dose more than 2 weeks earlier. These individuals were not expected to have developed significant immunity from COVID-19 at the point they were used as controls. We then matched the vaccinated cases to the set of suitable controls separately for each day of the follow-up period.

A requirement for vaccination was that individuals should not have had a COVID-19 infection in the previous 2 weeks. Therefore, as the rollout of the vaccination progressed and the population available as potential controls reduced through vaccination, the group not yet vaccinated and available as potential controls contained a progressively higher proportion of people who tested positive for COVID-19. This selection process biases the rate of positive tests in the control group upwards and would artificially inflate estimated vaccine effectiveness. To correct for this bias, we sequentially adjusted event rates in the intervention and control groups so that they remained consistent in the first 11 days of follow-up. Appendix 2 documents the adjustment method used.

### Matching and statistical analysis

**Fig. 2:**
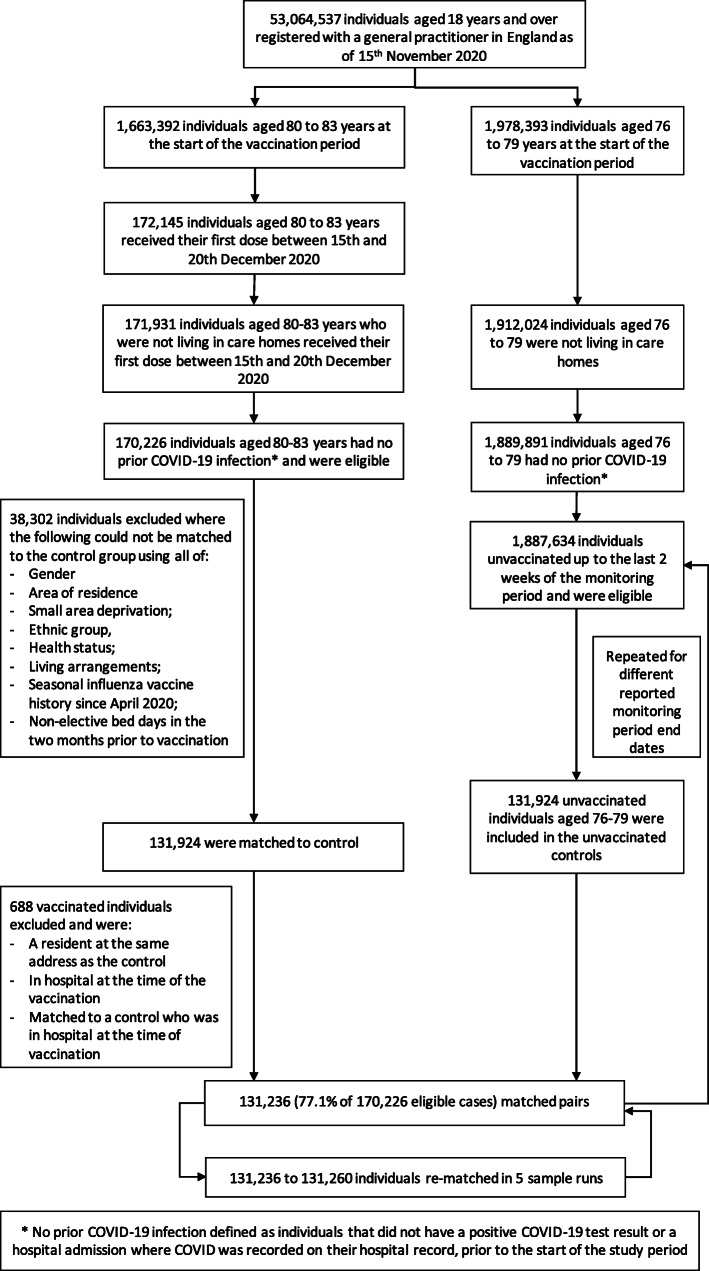
Flow diagram of the study population with eligibility criteria, exclusions, and matching methodology

Isolating the impact of vaccination requires a study design that accounts for temporal changes in infection rates, for which we used 1:1 exact matching [13] to account for several factors associated with exposure and outcomes: gender; area of residence [14]; small area deprivation [15]; ethnic group; health status; living arrangements; seasonal influenza vaccine history since April 2020; and emergency hospital stays in the previous 2 months. We excluded 1705 (1.0%) individuals with prior COVID-19 history to avoid likely pre-existing immunity [16, 17]. We excluded individuals from the control group if they were living in care homes or were not alive on the 15 December 2020. We dropped matched pairs where either individual was in hospital on the vaccination date or the pair lived at the same property (see Fig. 2).

We matched vaccinated individuals to unique controls without replacement. We assigned a control randomly where multiple matches were available for a vaccinated individual. We repeated the matching process five times with different random number seeds to create five matched populations. We bootstrapped 100 samples with replacement from each of these five matched populations and obtained confidence intervals using percentile values from the 500 samples.

### Tests of robustness

To explore the robustness of the adjustment for selection bias in the control group and the sensitivity of the results to the age group used for the controls, we compared the older age group to two different younger age groups. First, we matched vaccinated individuals aged 80–81 to controls aged 76–77 and vaccinated individuals aged 82–83 to controls aged 78–79. Second, we matched vaccinated individuals aged 80–81 to controls aged 72–73 and vaccinated individuals aged 82–83 to controls aged 74–75. The younger control group is less similar in age but unexposed to the vaccine for longer and therefore less prone to selection bias. We also tested the sensitivity of the results to reducing the inclusion criteria for eligible controls from 2 to 1-week post first vaccine dose (see Appendix 6). We used the STROBE (Strengthening The Reporting of OBservational Studies in Epidemiology) cohort checklist when writing our report [18].

## Results

### Study population

Of the total 1,685,530 individuals aged 80–83, 170,226 met our inclusion criteria. They were not residents of care homes, had no prior history of COVID-19, and received a first dose of the BNT162b2 mRNA vaccine between 15 and 20 December 2020 (Fig. 2). Of these, we exact-matched 131,236 (77.1%) to control individuals aged 76–79 who were not yet eligible for vaccination (Figs. 1 and 2). The requirement for an exact match generated a matched study population with lower proportions of individuals who were frail or clinically extremely vulnerable, from minority ethnic groups, or from socially-deprived areas compared to the full study population (Table 1 and Appendix 4).

**Table 1:**
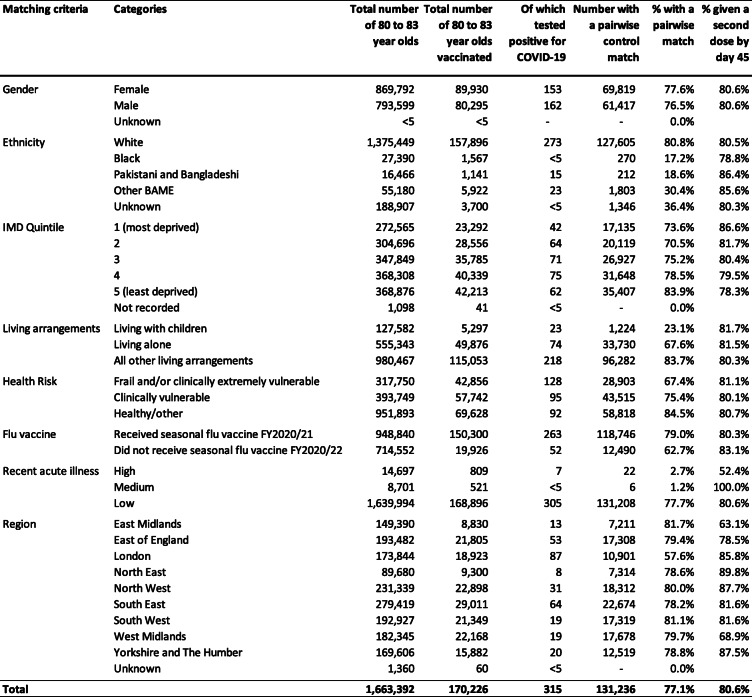
Demographic and clinical characteristics of vaccinated persons and their unvaccinated controls based on the matched cohort at baseline (day 11 after vaccination)

*BAME* Black, Asian and Minority Ethnicity, *IMD* Index of Multiple Deprivation, *FY2020/2021* Financial Year 2020/2021, running from 1 April 2020 to 31 March 2021

### Vaccine effectiveness

Across 45 days of follow-up, there was an average of 13.7 documented SARS-CoV-2 infections per day per 100,000 vaccinated individuals, compared to 23.2 per 100,000 unvaccinated controls. Over the same period, a daily average of 5.0 individuals per 100,000 attended an emergency department with COVID-19 and 5.3 per 100,000 were hospitalised with COVID-19 amongst the vaccinated cohort, compared to 9.6 per 100,000 (attended) and 9.4 per 100,000 (hospitalised) amongst unvaccinated controls.

**Fig. 3:**
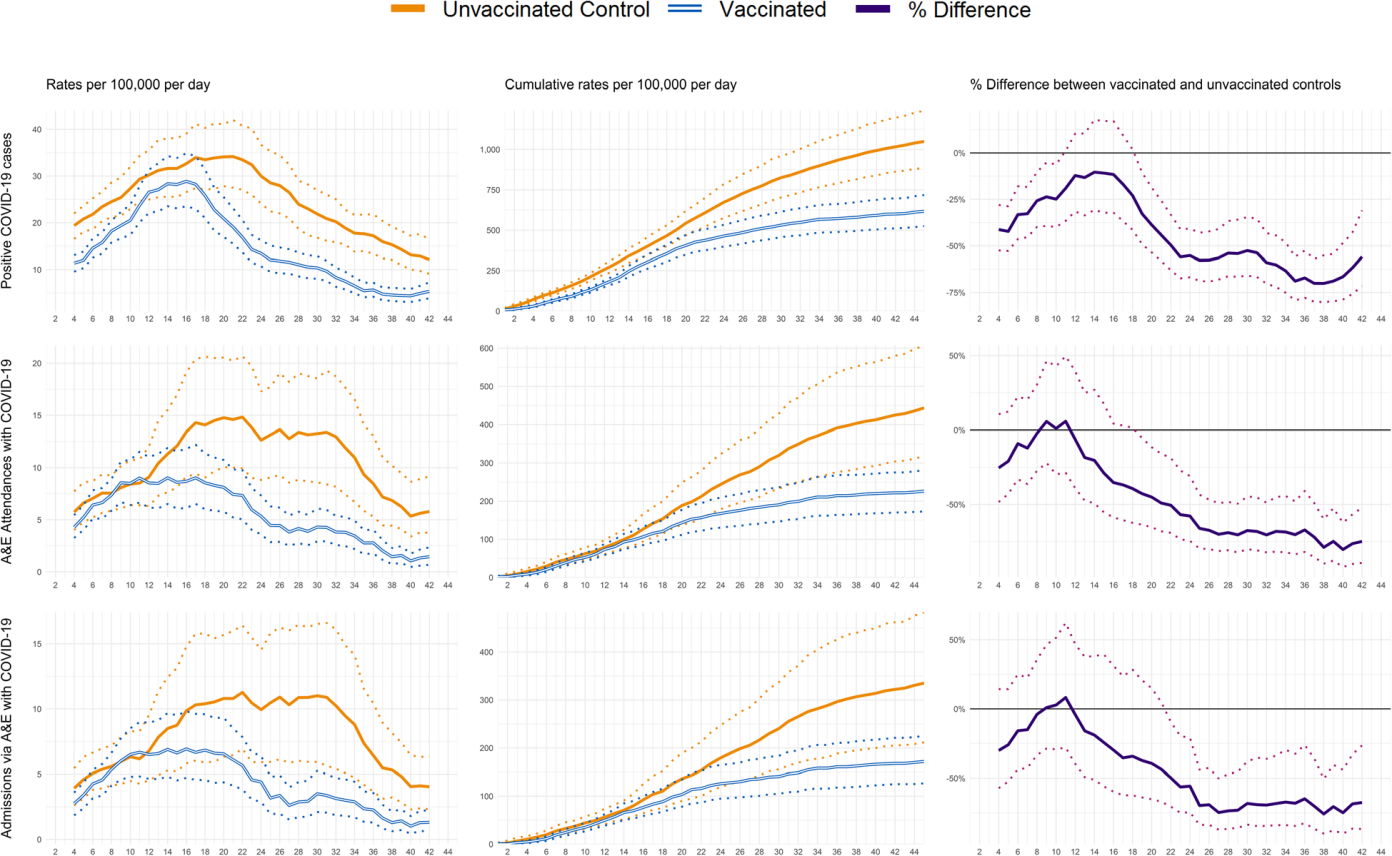
Profiles of positive COVID-19 infections, emergency department (A&E) attendances, and unplanned hospital admissions by days since first dose of vaccination. The data represent people aged between 80 to 83 years who received their first dose of the BNT162b2 mRNA COVID-19 vaccine between the 15 and 20 December 2020 with comparison to their matched controls. 95% confidence intervals are displayed as dashed lines

For the unvaccinated comparison group, COVID-19 event rates increased in the first 2 weeks of follow-up, with documented infections reaching a maximum at day 20, and emergency hospital attendances and admissions peaking between days 23 and 26 (Fig. 3). These profiles reflect the shape of the COVID-19 pandemic in England where prevalence peaked around the 1 January 2021 [19], and hospitalisations peaked in the second week of January 2021 [20]. For the vaccinated group, documented infections peaked earlier (day 14) and hospitalisations peaked between days 23 and 26.

We found similar results when we matched vaccinated individuals to unvaccinated individuals aged 72–75 years and when the comparison group was restricted to individuals who remained unvaccinated throughout the follow-up period (see Appendix 6).

Table 2 shows vaccine effectiveness, defined as percentage difference between vaccinated and unvaccinated groups, for each outcome across four time periods. Effectiveness increased over the follow-up period for all three outcomes. Estimated effectiveness at 21–27 days was 55.2% (95% CI 40.8–66.8%) for documented infection, 57.8% (30.8–74.5%) for emergency hospital attendances, and 50.1% (19.9–69.5%) for admissions. By days 35–41, the estimated effectiveness was 70.1% (55.1–80.1%) for documented infection, 78.9% (60.0–89.9%) for emergency department attendances, and 75.6% (52.8–87.6%) for hospitalisations.

**Table 2:**
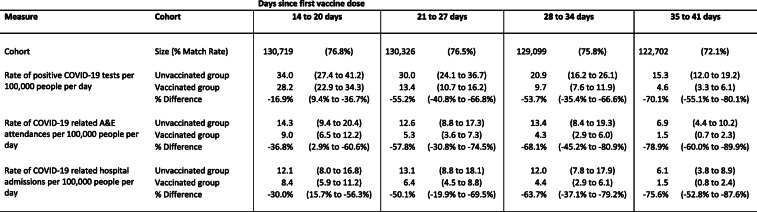
Estimates of the effectiveness of the BNT162b2 mRNA COVID-19 vaccine by days since vaccination

## Discussion

We considered 171,931 individuals aged 80 to 83 years in England who received a first dose of BNT162b2 mRNA COVID-19 vaccine as part of the nationwide NHS vaccination campaign in England. We compared their rates of SARS-CoV-2 positive tests and COVID-19 hospitalisations in the subsequent 45 days to those for slightly younger individuals with the same characteristics who became eligible for vaccination later. Emergency admission was 50.1% (19.9 to 69.5%) less likely 21 to 27 days after vaccination and 75.6% (52.8 to 87.6%) less likely 35 to 41 days after first vaccination and 7 days after 80% had received their second dose. COVID-19 infection was 55.2% (40.8 to 66.8%) less likely 21 to 27 days after vaccination and 70.1% (55.1 to 80.1%) less likely 35 to 41 days after first vaccination and 7 days after 80% had received their second dose. Collectively, these results are consistent with one dose of the BNT162b2 mRNA vaccine reducing events from 14 days after vaccination, with more effectiveness in reducing the severity of symptoms than preventing infection.

Our results are broadly consistent with existing estimates of BNT162b vaccine effectiveness, despite variations in study design, participant demographics, and outcome definitions [21]. We estimate effectiveness against documented infection of approximately 55% 21–27 days after one dose, rising to 70% after the majority received a second dose. These estimates are relatively consistent with results from a similar studies in England (55% after one dose, 80% 7 days after all received a second dose) [8] and Scotland (78% 21–27 days after one dose) [7] and from the older age group in a similar study in Israel (50% after one dose, 95% 7 days after all received a second dose) [5]. These estimates are also comparable to estimates of vaccine effectiveness against documented infection in working-age adults in England (70% after one dose, 85% 7 days after second dose) [9] and to all-age results from a randomised controlled trial (52% rising to 95%) [3]. Our point estimate of effectiveness against hospitalisation with COVID-19 (76% 7 days after most received a second dose) is somewhat lower than, though statistically compatible with, other estimates (80–87%) [5, 8].

Several factors likely contribute to these differences. First, our estimates are specifically for an older population where vaccine-induced immune responses may be sub-optimal [12]. In addition, our population included 20% of vaccinated individuals for whom the second dose was extended beyond the study period. This may explain the greater agreement with existing estimates for effectiveness 21–27 days after vaccination than for longer follow-up when second dose coverage varied between studies. However, a study based in Scotland where the majority received only single dose BNT162b estimated 87% (70 to 94) effectiveness against hospitalisation in those aged 80 and over at an equivalent time point (35–41 days post vaccination) [7], suggesting that differences in second dose coverage may not explain the differences between estimated effectiveness.

Finally, an important consideration in observational studies is bias in selection into the intervention group. While all existing studies used statistical methods to adjust for biases [5, 7, 8, 21], we exploited the precise age thresholds that determined temporal eligibility for vaccination, thereby reducing the risk of unmeasured confounding between cases and controls. Such biases are exacerbated with longer follow-up periods as those remaining unvaccinated become increasingly different from those vaccinated earlier. The divergence between our effectiveness estimates and those in other studies with longer follow-up may reflect less bias in our study design and adjustment methodology.

We focused on older people at high risk of serious COVID-19 outcomes. We considered a period and country experiencing widespread transmission and large numbers of hospitalisations. This provided statistical precision in the effectiveness estimates within a short period. We exploited a precise age cut-off that determined access to the vaccine, which reduced bias from selection into treatment.

Nonetheless, there is a risk of bias from unmeasured confounding with any observational study. We matched cases and controls on combinations of 12 personal, household and area variables. We also compared four measures of hospital use in the previous 18 months and history of negative SARS-CoV-2 tests (see Appendix 5). Vaccinated individuals did not have lower event rates and had higher use of hospital services and more community-based COVID-19 tests prior to vaccination when compared to the control group. This likely reflects the age difference which may bias our estimates towards lower than true effectiveness.

The rich set of matching variables meant some cases were excluded because there was no control available. These exclusions were more likely for some populations, including minority ethnic groups and residents of London, but the included individuals had similar outcomes to the excluded individuals and the effectiveness results were similar when we matched on fewer variables (Appendix 4).

The speed of the rollout of the NHS vaccination programme in England into younger populations reduced the pool of similar people who had not been vaccinated. We adjusted for the selection bias this generated and assessed the robustness of this adjustment by comparing to a younger age group where the selection bias occurred later in the monitoring period.

Finally, we considered COVID-19 related hospitalisations as well as positive COVID-19 tests. Hospitalisations are less likely to be influenced by changes in attitudes after receiving a vaccine that may affect whether individuals seek COVID-19 tests, such as misperceptions of immunity or misinterpretations of symptoms as side effects.

## Conclusions

We provide evidence of high real-world effectiveness of the original dosing schedule of the BNT162b2 mRNA COVID-19 vaccine in preventing infections and hospitalisations despite the widespread transmission of the B.1.1.7 variant shortly after the study population was vaccinated. There have been concerns about reduced vaccine effectiveness, though our data is consistent with mass vaccination data [5, 7, 8] and only slightly reduced neutralisation of B.1.1.7 pseudovirus relative to the Wuhan reference strain [22].

Our study provides rigorous evidence to support effectiveness of vaccination in the real-world amongst older people. Future research priorities include the optimal dosing regimen, the longevity of this protection and applicability to other variants, effectiveness amongst younger people and specific population subgroups, and effects on onward transmission and asymptomatic infection.

## Supplementary Information

**Additional file 1: Appendix 1**: Data sources. **Appendix 2**: Matching and adjustment methodology: Table A2-1 - Summary of the change in the number and COVID-19 status of people available for matching to vaccinated individuals as the monitoring period used in the evaluation is extended. Figure A2-1 - Numbers of individuals testing positive for COVID-19 post vaccination with a comparison to their match pairs as a rate per 100,000 pre and post adjustment. **Appendix 3**: Measuring COVID-19 related emergency hospital attendances and admissions: Table A3-1 - List of clinical codes used to identify first COVID-19 related ED attendances and hospital admissions. Figure A3-1 - Delay between specimen data for a COVID-19 positive test and the associated ED (A&E) attendance. Figure A3-2 - Counts of COVID-19 related ED (A&E) attendances and admissions by method of identification, for (a) attendances, and (b) admissions. **Appendix 4**: Changing composition of the study population by follow-up period: Table A4-1 - Demographic and clinical characteristics of vaccinated persons and their unvaccinated controls. **Appendix 5**: Comparison of vaccinated and unvaccinated controls pre-vaccination programme: Table A5-1. Adjusted odds ratios generated using a logistic regression model to predict test positivity between days 14 and 41 post vaccination event for vaccinated individuals and their pairwise controls. Figure A5-1 - Comparison of the use of hospital-based services per day for the vaccinated and the unvaccinated pairwise control group. Figure A5-2 - Comparison of the number of negative COVID-19 tests by specimen date for the vaccinated group and unvaccinated control. **Appendix 6**: Sensitivity of outcomes to control selection: Figure A6-1 - Percentage difference in positive COVID-19 tests, ED (A&E) attendances with COVID-19, hospital admission with COVID-19 for six matching strategies by day since first vaccine dose. Table A6-1 - Comparison of estimates of the effectiveness of the BNT162b2 mRNA Covid-19 vaccine by days since vaccination for six matching strategies. References for supporting appendices

## Data Availability

Data used in the study are not publicly available and may be obtained via NHS Digital (https://digital.nhs.uk/services/data-access-request-service-dars). The analysis scripts used for the evaluation are available at https://github.com/NHSEI-Analytics/nhs_covid19_effectiveness.

## Abbreviations

A&E: Accident and Emergency (also known as Emergency Department)
APC CDS: Admitted Patient Care Commissioning Dataset
BAME: Black, Asian and minority ethnic
BNT162b2: BioNTech 162b2 [vaccine]
COPI: Control of patient information
COVID-19: Coronavirus disease 2019
ECDS: Emergency Care Dataset
ED: Emergency department
FY: Financial Year
ICD-10: International Classification of Diseases 10th Revision
ICMJE: International Committee of Medical Journal Editors
IMD: Indices of multiple deprivation
MPI: Master Patient Index
mRNA: Messenger ribonucleic acid
MSOA: Middle layer super output area
NHAIS: National Health Application and Infrastructure Services
NHS: National Health Service
NIHR: National Institute for Health Research
NIMS: National Immunisation Management Service
ONS: Office for National Statistics
PCR: Polymerase chain reaction
SARS-CoV-2: Severe acute respiratory syndrome coronavirus 2
SNOMED CT: Systematized Nomenclature of Medicine Clinical Terms
STROBE: Strengthening The Reporting of OBservational Studies in Epidemiology
