# Supplementary material for: Effects of BNT162b2 mRNA vaccine on COVID-19 infection and hospitalisation amongst older people: matched case control study for England
Source: BMC Med. 2021 Oct 18;19:275. doi: 10.1186/s12916-021-02149-4 (PMC8523007; doi:10.1186/s12916-021-02149-4)
Supplement: Supplementary file 1 — Additional file 1: Appendix 1: Data sources. Appendix 2: Matching and adjustment methodology: Table A2-1 - Summary of the change in the number and COVID-19 status of people available for matching to vaccinated individuals as the monitoring period used in the evaluation is extended. Figure A2-1 - Numbers of individuals testing positive for COVID-19 post vaccination with a comparison to their match pairs as a rate per 100,000 pre and post adjustment. Appendix 3: Measuring COVID-19 related emergency hospital attendances and admissions: Table A3-1 - List of clinical codes used to identify first COVID-19 related ED attendances and hospital admissions. Figure A3-1 - Delay between specimen data for a COVID-19 positive test and the associated ED (A&E) attendance. Figure A3-2 - Counts of COVID-19 related ED (A&E) attendances and admissions by method of identification, for (a) attendances, and (b) admissions. Appendix 4: Changing composition of the study population by follow-up period: Table A4-1 - Demographic and clinical characteristics of vaccinated persons and their unvaccinated controls. Appendix 5: Comparison of vaccinated and unvaccinated controls pre-vaccination programme: Table A5-1. Adjusted odds ratios generated using a logistic regression model to predict test positivity between days 14 and 41 post vaccination event for vaccinated individuals and their pairwise controls. Figure A5-1 - Comparison of the use of hospital-based services per day for the vaccinated and the unvaccinated pairwise control group. Figure A5-2 - Comparison of the number of negative COVID-19 tests by specimen date for the vaccinated group and unvaccinated control. Appendix 6: Sensitivity of outcomes to control selection: Figure A6-1 - Percentage difference in positive COVID-19 tests, ED (A&E) attendances with COVID-19, hospital admission with COVID-19 for six matching strategies by day since first vaccine dose. Table A6-1 - Comparison of estimates of the effectiveness of the BNT162b2 mRNA Covi [file 12916_2021_2149_MOESM1_ESM.docx]

**Supplementary Appendices: Effects of BNT162b2 mRNA vaccine on Covid-19 infection and Hospitalisation among older people: matched case control study for England**

**Appendix 1: Data sources**

**Appendix 2: Matching and adjustment methodology**

**Appendix 3: Measuring COVID-19 related emergency hospital attendances and admissions**

**Appendix 4: Changing composition of the study population by follow-up period**

**Appendix 5: Comparison of vaccinated and unvaccinated controls pre-vaccination programme**

**Appendix 6: Sensitivity of outcomes to control selection**

**Appendix 1: Data sources**

NHS England & Improvement has access to person-level datasets for the entire population of England that provide information on who has been vaccinated, by date, vaccine type and dose, their age, gender and details of the address they live at, if they have had a prior COVID-19 infection (that is reported) and whether they go on to test positive for COVID-19 post vaccination. These datasets, that are collated by NHS Digital, Public Health England and the Office for National Statistics, include information on the contacts individuals had with the health service before and since being vaccinated, including Emergency Department (ED) attendances (also known as Accident & Emergency (A&E) attendances) and hospital admissions, and information regarding people who have since died. In combination, these datasets offer significant insights into what happens to people post their first vaccination dose to understand the effectiveness of the vaccines.

A Master Patient Index (MPI) data mart has been developed that includes details of all NHS registered patients in England. The MPI is built from extracts from the National Health Application and Infrastructure Services (NHAIS) system^1^, and comprises a list of NHS registered patients including details of their gender, age, area of residence together with a variety of derived data items including whether they are a permanent resident of a care home (based on address matching to registered care home details from the Care Quality Commission), detail of house occupancy/living arrangements (of which three categories have been used for the pairwise matching methodology: living with children under 18 years, living alone, and all other living arrangements) and supplementary information sourced from the Indices of Multiple Deprivation (IMD) classification for 2019.^2^

Several data assets have been linked to the MPI based on NHS Number (using a common pseudonym within a secure environment in accordance with the Control of Patient Information (COPI) notice^3^) including:

- Vaccinations event data sourced from the National Immunisation Management Service (NIMS)^4^ to provide details of the 1st and 2nd doses of the BNT162b2 mRNA and the ChAdOx1 adenovirus vector vaccines at person level. The extracts taken from the NIMS system undergo a series of transformations and data cleaning steps to identify events where vaccinations were given in line with those used for publication^5^. The dataset is also used to identify people who received a seasonal flu vaccine during FY2020/21;
- Hospital-based (Pillar 1) and community-based (Pillar 2) COVID-19 positive and negative tests data sourced from Public Health England Unified Sample Dataset^6^. For this analysis a dataset prepared by PHE that provides details of the 1st positive polymerase-chain-reaction (PCR) COVID-19 test per individual is used;
- Death registrations sourced from the Office for National Statistics (ONS)^7^;
- Emergency Department (A&E) attendance records sourced from the Emergency Care Dataset (ECDS) via NHS Digital^8^;
- Admitted patient care hospitals spell records sourced from the Admitted Patient Care Commissioning Dataset (APC CDS)^9^ via NHS Digital’s SUS+ Service^10^; and
- Shielded Patient List as generated by NHS Digital using a series of patient level collections to identify clinically extremely vulnerable individuals.^11^

A number of derived data items have been incorporated into the data mart to provide information on the health status of individuals, including the ‘Bridges to Health’ segmentation model^12^ (as adapted by Outcomes Based Healthcare^13^ to use data for the English health care system) that identifies patients with co-morbidities based on their hospital records that increase their risk of hospitalisation (here referred to as clinically vulnerable), and an algorithm applied to historic APC CDS spells data to identify frail individuals^14^. For the pairwise matching methodology, these data items have been combined into a measure of health status comprising three categories: frail and/or clinically extremely vulnerable, clinically vulnerable, and other/unknown (which predominately represents relatively healthy individuals).

Individual-level ethnicity data sourced from the APC CDS^9^, ECDS^8^, Outpatient Commissioning Dataset^15^, Mental Health Services Dataset^16^ and Community Services Dataset^17^ have been supplemented by data from NHS Digital sourced from a range of administrative sources include General Practice records to generate an ethnicity register. For the pairwise matching methodology four categories have been used: White, Black, Pakistani, or Bangladeshi, another ethnicity, and ethnicity not reported. These categories are correlated with vaccine uptake in the wider population^18^.

A weighted acute illness measure has been derived using completed APC CDS spells. Total non-elective occupied bed days for individuals discharged between 15^th^ November and 14^th^ December have been multiplied by two, and the equivalent measure for discharges between the 15^th^ October 2020 and 14^th^ November 2020 remain unadjusted, with the two values summed. Spells where an individual was admitted and discharged on the same day count as 0.5 bed days. The acute illness measure is then defined as follows: high non-elective bed use (6 plus weighted bed days); medium non-elective bed use (3 to 5 weighted bed days), and low/no non-elective bed use (0 to 2 weighted bed days).

The refresh cycle for the data sources used in this data mart are daily for the vaccinations, testing, mortality, ED and APC datasets. The MPI is refreshed on a monthly cycle. The coverage, completeness and quality of these collections vary, and steps have been taken to ensure the data used in the evaluation are complete. The data mart used to generate the results presented in this analysis was extracted on the 9^th^ February 2021 and includes complete records to the 3^rd^ February 2021. This uses a cut of the MPI from mid-November 2020 and all age-based calculations reference individuals ages as of mid-November 2020.

**Appendix 2: Matching and adjustment methodology**

We matched vaccinated individuals in their early 80s to controls in their late 70s. This exploits the age-based eligibility criteria for the nationwide population vaccination^19^, whereby people aged 80 years and over were prioritised for vaccination. Whilst this approach helps minimise these biases in the early stages of the vaccination programme, from mid-January 2021 significant numbers of 70-year-olds had received their 1^st^ vaccine dose. Because we exclude individuals who were vaccinated more than 14 days before the end of the monitoring period from the pool of potential controls, this generates bias whereby the proportion of people in the pool available for matching becomes enriched in people who test positive for COVID-19 as individuals should not have had a COVID-19 infection in the two weeks prior to vaccination. If unaccounted for, this selection bias artificially increases the number of COVID-19 positive people in the pairwise control relative to the vaccinated cohort (see Table A2-1).

**Table A2-1.** Summary of change in number and COVID-19 status of people available for matching to vaccinated individuals as the monitoring period used in the evaluation is extended. The analysis has been run twice: once excluding controls that received their first COVID-19 dose during the monitoring period, and a second time excluding controls that received their first COVID-19 dose before 14 days of the end of the monitoring period (which is the approach used in the main analysis).

To adjust for this bias, we have developed a methodology where we generate a number of daily timeseries for each outcome by extending the monitoring period a day at a time, starting with an end date 11 days post vaccination (at which point the bias is minimal) and repeating the process 35 times to extend the monitoring period to 45 days post vaccination. With each iteration the size of the potential pool of match pairs contracts as we exclude from the pool of potential controls individuals who were vaccinated more than 14 days before the end of the monitoring period.

We then align each timeseries so that vaccination events on the 15^th^, 16^th^, 17^th^, 18^th^, 19^th,^ and 20^th^ December 2020 reference to day 0 with up to 45 days follow-up. The absolute numbers for each outcome (positive tests, ED attendances with COVID-19 and admissions with COVID-19 via ED) to hospital) are converted into rate per 100,000 per day by dividing by the size of the matched cohort for each of the 35 runs.

Next, we compare the total cumulative rate for each run to the previous day’s run excluding the latest day from the former to calculate the change run-on-run (a_d_) as below:

$$a_{d}=\frac{\sum_{t=1}^{d-1} y_{t,d}^{c}}{\sum_{t=1}^{d-1} y_{t,d-1}^{c}}$$

where $y_{t,d}^{c}$ the event rate per 100,000 persons at time t for the control group, c represents the control cohort, $t$ is the start date of the monitoring period (which is fix as Day 1), and *d* is the last day for each run. This generates a set of values for *a_d_* that are applied to correct for the cumulative sampling bias using as follows:

$$\tilde{y_{t,d}^{c}}=y_{t,d}^{c}.\frac{1}{\prod_{d=1}^{d} a_{d}}$$

Following these steps, we generate a set of adjusted time series for each run for the controls and vaccinated cohorts as illustrated in Figure A2-1. For each run we take the latest value as the best estimate of the adjusted daily figure for that point in time as denoted by the dashed line.

The process is repeated five times for five separate batches (where the matching process is repeated with different random number seeds, allowing replacement in match-pairs between the sensitivity runs). A simple average is taken across the five batches as follows:

$$y_{t,d}^{c}=\frac{1}{N}\sum_{n=1}^{N} y_{t,d,n}^{c}$$

We then run a bootstrapping procedure with replacement 100 times within each of the batches. This gives us 5x100 values for each of the key statistics (event rate in vaccinated, event rate in matched controls, relative reduction in event rate). We use the distribution of these statistics to generate 95% confidence intervals (by picking the 12th (0.025*500) lowest and highest values). For graphical purposes only, seven-day moving averages of the rates are calculated and presented.

**Figure A2-1.** Numbers of individuals testing positive for COVID-19 post vaccination (in blue) with a comparison to their match pairs (in orange) as a rate per 100,000. The left-hand charts present the raw numbers pre-adjustment for the sampling bias where each line represents to a different cut-off date for the analysis that varies the size of the pool of individuals available for matching. The right-hand charts present the adjusted figures based on the methodology, where the dashed blue and dashed orange lines represent the most complete estimate by day since vaccination.

**
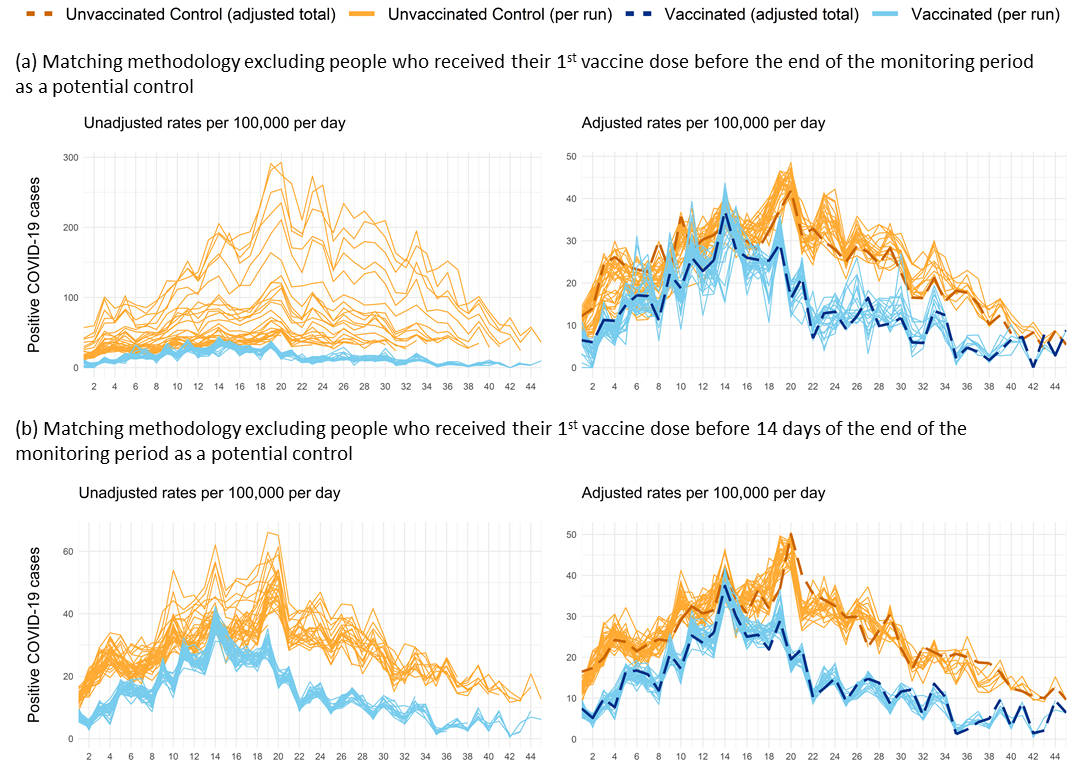
**

**Appendix 3: Measuring COVID-19 related emergency hospital attendances and admissions**

Two outcome measures of hospitalisation with COVID-19 have been used in the evaluation: (1) Emergency Department (ED) attendance with Covid-19 measured using the Emergency Care Data Set (ECDS), and (2) non-elective admission to hospital measured using the Admitted Patient Care Commissioning Dataset (APC CDS). Both datasets have timeliness issues with coverage and coding completeness that can bias analysis if not accounted for.

To circumvent issues with coding completeness, a matching algorithm has been used to identify COVID-19 related ED attendances and admissions by combining diagnosis information available from the ECDS/APC records (see Table A3-1) with COVID-19 positive test results where the specimen was taken between 14 before and 6 days after the linked ED attendance/admission. Using this matching window, 23% of linked test results were taken between 14 and 1 days before the ED attendance, with 58% having a linked tests taken on days 0 to 1 post attendance, and 19% having a linked test taken between days 2 and 6 days post attendance (see Figure A3-1). Coding completeness is less of an issue with the APC CDS data, where most COVID-19 related admissions both have a COVID-19 diagnosis on the APC record, and a linked COVID-19 positive test result (see Figure A3-2).

**Table A3-1.** List of SNOMED CT (Systematized Nomenclature of Medicine Clinical Terms) and ICD-10 (International Classification of Diseases 10th Revision) codes used to identify first COVID-19 related ED attendances and hospital admissions based on the primary diagnosis, secondary diagnoses, and (for ECDS) notifiable diseases fields within the ECDS and APC CDS collections. Several additional SNOMED CT codes are available for certain conditions that present with COVID-19, but an analysis suggest none of these codes (and other associated codes for COVID-19) have been used within the ECDS collection.

| **SNOMED CT Code/**  **ICD-10 Code** | **Description** |
| --- | --- |
| 1240751000000100 | Coronavirus disease 19 caused by severe acute respiratory syndrome coronavirus 2 (disorder) |
| 1240761000000102 | Suspected coronavirus disease 19 caused by severe acute respiratory syndrome coronavirus 2 (situation) |
| 1300721000000109 | Coronavirus disease 19 caused by severe acute respiratory syndrome coronavirus 2 confirmed by laboratory test (situation) |
| U07.1 | COVID-19, virus identified |
| U07.2 | COVID-19, virus not identified |
| B97.2 | Coronavirus as the cause of diseases classified to other chapters |

**Figure A3-1.** Delay between specimen data for a COVID-19 positive test and the associated ED (A&E) attendance for 80- to 83-year-olds vaccinated between the 15^th^ and 20^th^ December 2020.


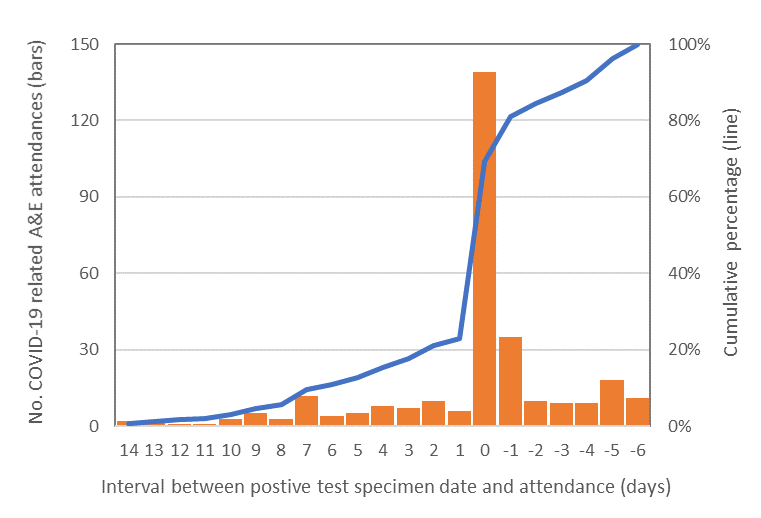


**Figure A3-2.** Counts of COVID-19 related ED (A&E) attendances and admissions for 80- to 83-year-olds vaccinated between the 15^th^ and 20^th^ December 2020 by method of identification, for (a) attendances, and (b) admissions.

**
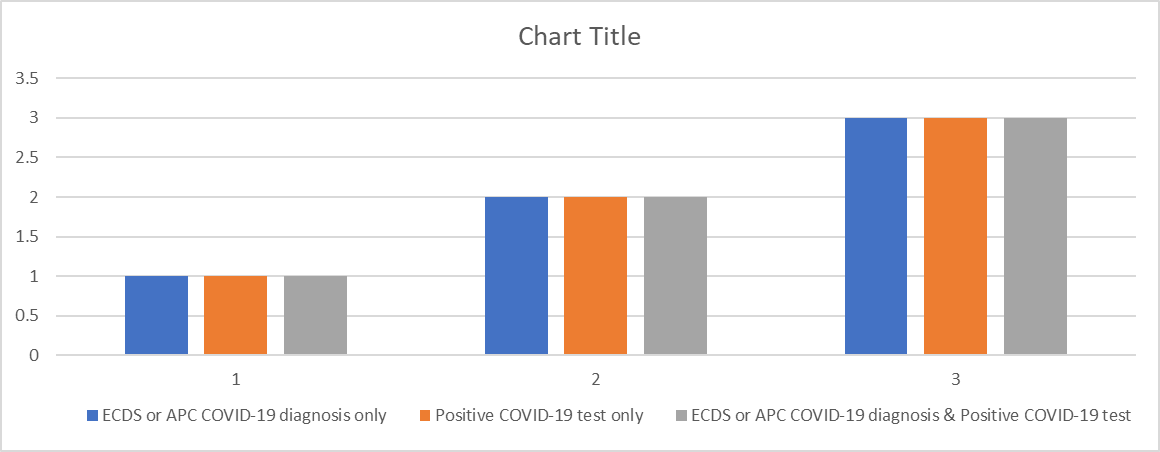
**
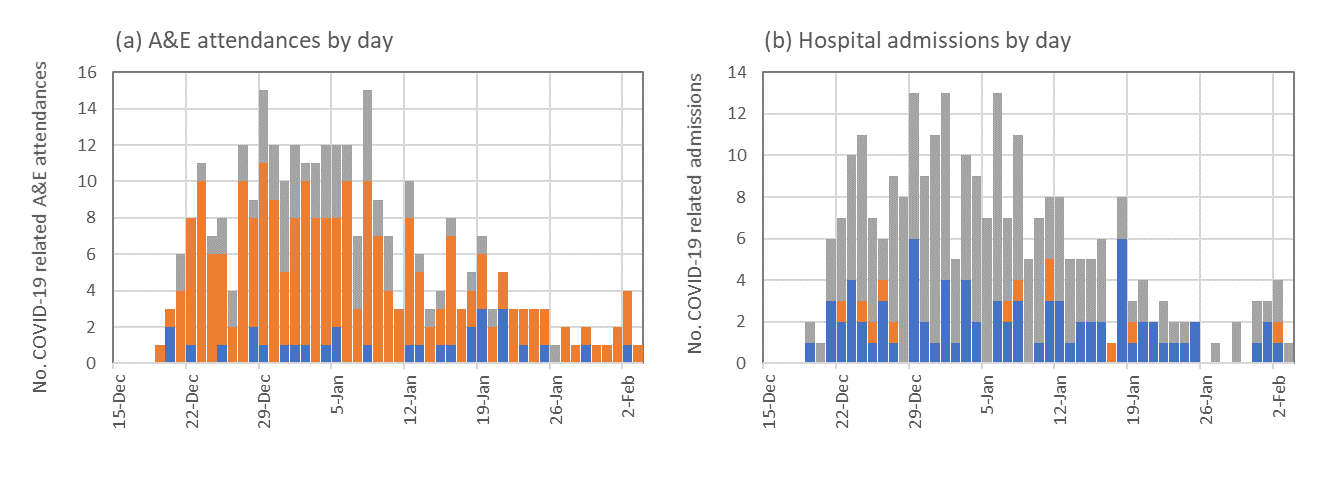


The matching algorithm allows all ED attendances and/or admissions within this matching window to be counted as COVID-19 related, or it can be constrained to select the first COVID-19 related attendance and/or admission per person. Using the latter approach avoids counting multiple attendances and/or admissions for the same person and is the approach used in this analysis.

For the ACP CDS, an additional complication relates to the collection being discharge centric whereby providers usually only submit records for patients that has been discharged from hospital. To allow reporting by admission date, we restricted the analysis to patients who stayed in hospital 42 days or less. This excludes 5.7% of COVID-19 related spells for 80- to 83-year olds (based on patients discharged between the 8^th^ December 2020 and the 31^st^ March 2021).

To account for incomplete coverage of the ECDS collection, the analysis is based on ED data for a sample of 118 NHS providers in England that have complete data to the 9^th^ February 2021 based on an extract taken on the 9^th^ March 2021. Similarly, to account for incomplete coverage of the APC CDS collection, the analysis is based on APC CDS data for a sample of 124 NHS providers in England that have complete data to the 17^th^ March 2021 based on an extract taken on the 6^th^ April 2021. These two samples accounts for 95% of Type 1&2 ED attendances and 93% of admitted patient care spell for England. As the pairwise control matching methodology matches the vaccinated individual to a control from the same middle super output area, we do not expect a systematic bias in the analysis due to incomplete coverage.

These attendances and admissions are referred to as ‘with’ COVID-19 as the available data does not allow us to definitively determine if their COVID-19 infection was the primary reason for their ED attendance or admission to hospital.

**Appendix 4: Changing composition of the study population by follow-up period**

The adjustment methodology described in Appendix 2 involves re-matching vaccinated individuals to controls as the follow-up period is extended a day at a time. Therefore, depending on the length of follow-up, the size and composition of both the vaccinated group and unvaccinated control group change. For example, in the main analysis, the match rate falls from 77.0% at day 11 (which represents the baseline case) to 64.5% at day 45 (Table A2-1).

Table A4-1 presents the number and percentage of vaccinated individuals for whom a pairwise match was identified at the mid-points of each period of follow-up, stratified by each matching variable. While there is wide variability in the percentage of vaccinated individuals with a matched pair (from 1.2% to 84.2%) across subgroups at day 17 of follow-up, the relative composition of those included in analysis is relatively stable across extended follow-up times (Table A4-1). The largest changes were for health status category where the share of the health/other category increases by 0.41 percentage points, with concurrent reductions of 0.22 and 0.18 percentage points for the frail and/or clinically extremely vulnerable and clinical vulnerable groups respectively between 17 and 38. These changes are small and are unlikely to result in any significant bias to the effectiveness estimates as the monitoring period is extended.

Table A4-1 also provides unadjusted and adjusted estimates of cumulative documented infections amongst vaccinated and unvaccinated control individuals by subgroup. These were generated by selecting all records without replacement and running the same adjustment process that was used in the bootstrapping process in the main analysis. Note that the main analysis bootstrapping process sampled with replacement, so values differ from those in the main analysis. We derived the ratio of adjusted to unadjusted total infections and applied this as a normalisation factor to each subgroup to estimate adjusted subgroup counts.

**Table A4-1.** Demographic and clinical characteristics of vaccinated persons and their unvaccinated controls based on matching to the 76 to 79 years population. Four matched cohort at days 17, 24, 31 and 38 post vaccination are presented, which relate to the mid-points of each period of follow-up. Cumulative unadjusted and adjusted rates of COVID-19 positive tests are included to demonstrate differences in test-positivity rates between the subgroups.

**Table A4-1 (Cont.)** Demographic and clinical characteristics of vaccinated persons and their unvaccinated controls based on matching to the 76 to 79 years population. Four matched cohort at days 17, 24, 31 and 38 post vaccination are presented, which relate to the mid-points of each period of follow-up. Cumulative unadjusted and adjusted rates of COVID-19 positive tests are included to demonstrate differences in test-positivity rates between the subgroups.

**Appendix 5: Comparison of vaccinated and unvaccinated controls pre-vaccination programme**

The pairwise matching approach has been developed to include information on the socio-demographic characteristics of individuals who have been vaccinated, together with factors that are likely to be associated with individuals’ exposure risk to COVID-19 including the local prevalence of COVID-19 (as captured by the Middle Super Output Area (MSOA) of residence) and individuals living arrangements (with those living alone being more likely to have interactions with formal and informal carers). The matching approach also accounts for individuals’ susceptibility to developing COVID-19 and the severity of illness experienced, and factors associated with individuals’ behaviours that might predict their chances of being vaccinated.

A logistic regression model was used to test the statistical-significance of the parameters used in the pairwise matching approach for test positivity for COVID-19. Odd ratios for the regression are presented in Table A5-1. These results demonstrate that the majority of parameters used are significantly associated with individuals’ risk of developing COVID-19 during the monitoring period for the study.

**Table A5-1.** Adjusted odds ratios generated using a logistic regression model to predict test positivity between days 14 and 41 post vaccination event for vaccinated individuals and their pairwise controls. The model is based on data to day 41 post vaccination for 116,539 match pairs. p-values were set to 0.05 significance level, with further significance levels set to 0.01(*), 0.001(**) and <0.001 (***).

There may be other factors that are not accounted for by this matching approach that impact the outcomes being monitored. One approach for assessing if such unobserved biases are significant is to monitor outcomes for the intervention and control groups in the period before the intervention to assess how the intervention and control groups comparable. As the outcomes of interest for this evaluation are related to the first case of COVID-19 per person, we cannot do this directly. However, by comparing emergency hospital attendances, emergency hospital stays, outpatient attendances, and planned hospital stays over the period prior to the introduction of the vaccination programme for the vaccinated and control cohorts, we can assess how the matching approach performs in terms of healthcare utilisation (see Figure A5-1).

**Figure A5-1.** Comparison of the use of hospital-based services per day for the vaccinated group of 80 to 83 years olds (in blue) and the unvaccinated pairwise control group of 76 to 79 years olds (in orange) by activity type. Each data point reflects the daily total, and the solid lines present the seven-day centred moving averages. Data are sourced from the Emergency Care Dataset (ECDS), and the Outpatient and Admitted patient care datasets sourced from the SUS+ service and represent the baseline cohort of 131,236 match pairs at Day 11 post vaccination.


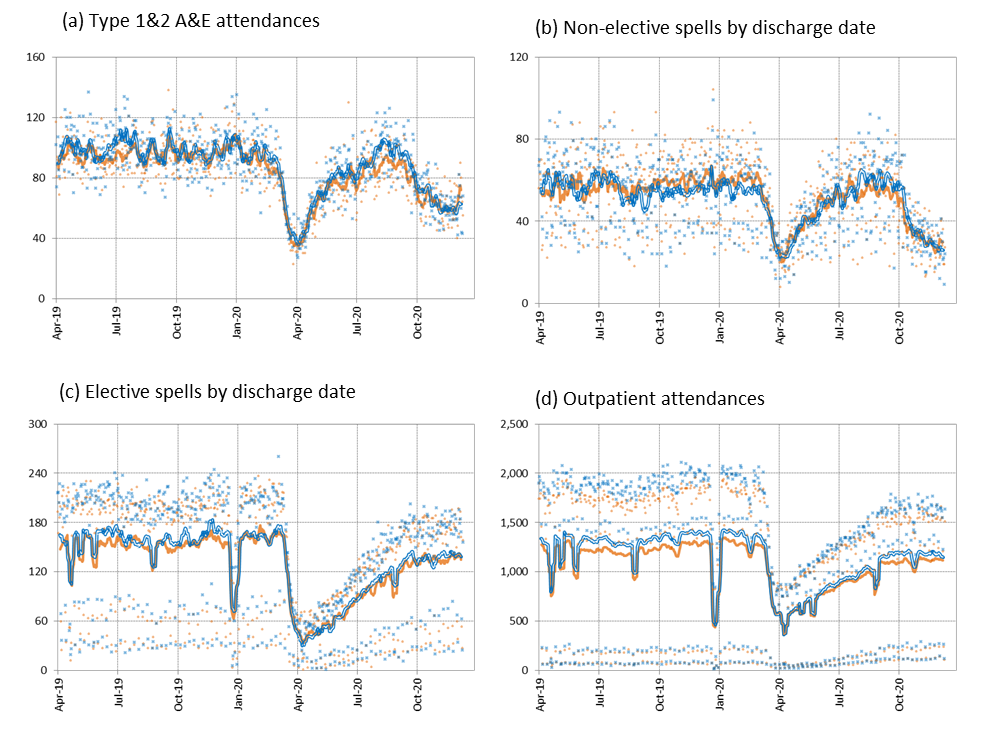


These results demonstrate a close match between the vaccinated and control cohorts in the 18 months to October 2020 for all four activity types. For planned care the vaccinated group is more likely to have used hospital-based health services in the previous 18 months, which is consistent with the vaccinated cohort being an average of 4 years older than their pairwise controls.

The number of negative tests undertaken for individuals within the vaccinated cohort and the control cohort are presented in Figure A5-2. As with the hospital activity measures, the number of negative tests undertaken is similar between the two groups, with the Pillar 1 negative tests (which represents those undertaken following contacts with the health system) showing a closer match when compared to Pillar 2 tests (which represent tests undertaken in the community) where the control group shows higher numbers of negative tests compared to the vaccinated group. This difference in Pillar 2 testing data is consistent with higher uptake rates of Pillar 2 tests by younger age groups, reflecting the average age difference of 4 years between the vaccinated and control groups.

**Figure A5-2.** Comparison of the number of negative COVID-19 tests by specimen date undertaken between the 1^st^ May and 30^th^ November 2020 for the vaccinated group and unvaccinated control. Each data point reflects the daily total, and the solid lines present the seven-day centred moving averages.


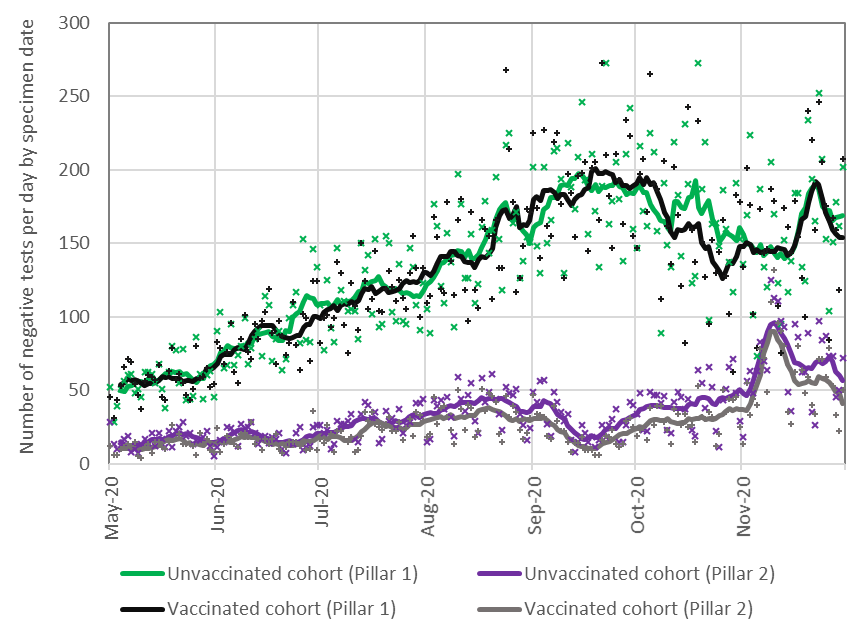


Overall, Figures A5-1 and A5-2 demonstrate a close match between the vaccinated and control groups before vaccination. These results, couple with the broad range of factors that are accounted for with the pairwise control matching process provides confidence the vaccinated control and matched controls are comparable and can be used to monitor outcomes.

**Appendix 6: Sensitivity of outcomes to control selection**

Due to the rapid vaccination rollout, controls who were ineligible for vaccination at the start of the follow-up period relatively quickly become eligible for vaccination (Figure 1 in the main text). This depleted the pool of available controls for matching at later stages of follow-up. In addition, because individuals must not have had documented infection in the two weeks before vaccination, those with documented infection during the study period became concentrated in the population of available controls. To adjust for this selection bias, we used a novel adjustment methodology (Methods & Appendix 2).

To test the sensitivity of our results to possible residual selection bias, we repeated analysis matching 80-83-year-old vaccinated individuals to 72-75-year-old unvaccinated controls, as this group became eligible for vaccination later than the control group used in the main analysis (Figure 1). We also tested the sensitivity of the results to (1) using only controls who remained unvaccinated throughout the follow-up period, and (2) limiting eligible of controls to within one week of 1^st^ vaccine dose compared to the two week inclusion criteria used for the main analysis. Finally, for hospital admissions we included an additional outcome measure for patients admitted via emergency departments based on a disposal code of admitted to hospital, transferred to another provider, or died as recorded in the Emergency Care Data Set.

Effectiveness estimates were broadly consistent whichever control population and selection criteria were used (see Figure A6-1 and Table A6-1). Effectiveness estimates were also consistent between the two measures of hospital admission.

**Figure A6-1.** Percentage difference in positive COVID-19 tests, ED (A&E) attendances with COVID-19, hospital admission via ED with COVID-19 and all <43-day length of stay hospital admissions with COVID-19 for six matching strategies by day since first vaccine dose. 95% confidence intervals are represented by the dashed lines.





**Table A6-1.** Comparison of estimates of the effectiveness of the BNT162b2 mRNA Covid-19 vaccine by days since vaccination for six matching strategies.

**References for supporting appendices**

1 National Health Application and Infrastructure Services (NHAIS). NHS Digital. https://digital.nhs.uk/services/nhais.html (accessed March 12, 2021).

2 Ministry of Housing Communities & Local Government. National Statistics: English indices of deprivation 2019. 2019. https://www.gov.uk/government/statistics/english-indices-of-deprivation-2019 (accessed March 11, 2021).

3 Control of patient information (COPI) notice. NHS Digital. https://digital.nhs.uk/coronavirus/coronavirus-covid-19-response-information-governance-hub/control-of-patient-information-copi-notice (accessed March 12, 2021).

4 National COVID-19 and Flu Vaccination Programmes. NHS England. https://www.england.nhs.uk/contact-us/privacy-notice/national-flu-vaccination-programme/ (accessed March 12, 2021).

5 COVID-19 Vaccinations. NHS England. <https://www.england.nhs.uk/statistics/statistical-work->areas/covid-19-vaccinations/ (accessed March 12, 2021).

6 COVID-19 testing data: methodology note 2020. Department of Health and Social Care. https://www.gov.uk/government/publications/coronavirus-covid-19-testing-data-methodology/covid-19-testing-data-methodology-note (accessed March 12, 2021).

7 Deaths. Office for National Statistics. https://www.ons.gov.uk/peoplepopulationandcommunity/birthsdeathsandmarriages/deaths (accessed March 12, 2021).

8 Emergency Care Data Set (ECDS). NHS Digital. https://digital.nhs.uk/data-and-information/data-collections-and-data-sets/data-sets/emergency-care-data-set-ecds (accessed March 12, 2021).

9 CDS V6-2 Type 130 - Admitted Patient Care - Finished General Episode CDS. NHS Data Dictionary. NHS Digital. https://datadictionary.nhs.uk/data_sets/cds_v6-2/cds_v6-2_type_130_-_admitted_patient_care_-_finished_general_episode_cds.html (accessed March 12, 2021).

10 Secondary Uses Service (SUS). NHS Digital. https://digital.nhs.uk/services/secondary-uses-service-sus (accessed March 12, 2021).

11 COVID-19 – high risk shielded patient list identification methodology. NHS Digital. https://digital.nhs.uk/coronavirus/shielded-patient-list/methodology (accessed March 12, 2021).

12 Lynn J, Straube BM, Bell KM, Jencks SF, Kambic RT. Using population segmentation to provider better health care for all: the “Bridges to Health” model. Milbank Q 2007; 85(2): 185-208.

13 Part 2: Whole Population Segmentation Models. Outcomes Based Healthcare. https://outcomesbasedhealthcare.com/bridges-to-health-segmentation-model/ (accessed March 12, 2021).

14 Gilbert T, Neuburger J, Kraindler J, et al. Development and validation of a Hospital Frailty Risk Score focusing on older people in acute care settings using electronic hospital records: an observational study. Lancet 2018; 391: 1775-1782.

15 CDS V6-2 Type 020 - Outpatient CDS. NHS Data Dictionary. NHS Digital. https://datadictionary.nhs.uk/data_sets/cds_v6-2/cds_v6-2_type_020_-_outpatient_cds.html#dataset_cds_v6-2_type_020_-_outpatient_cds.html (accessed August 24, 2021).

16 Mental Health Services Data Set. NHS Data Dictionary. NHS Digital. https://datadictionary.nhs.uk/data_sets/clinical_data_sets/mental_health_services_data_set.html#dataset_mental_health_services_data_set.html (accessed August 24, 2021).

17 Community Services Data Set. NHS Data Dictionary. NHS Digital. https://datadictionary.nhs.uk/data_sets/clinical_data_sets/community_services_data_set.html#dataset_community_services_data_set.html (accessed August 24, 2021).

18 Razai MS, Osama T, McKechnie DGJ, Majeed A. Covid-19 vaccine hesitancy among ethnic minority groups. BMJ 2021; 372: n513

19 Joint Committee on Vaccination and Immunisation: advice on priority groups for COVID-19 vaccination. Department for Health and Social Care. https://www.gov.uk/government/publications/priority-groups-for-coronavirus-covid-19-vaccination-advice-from-the-jcvi-30-december-2020/joint-committee-on-vaccination-and-immunisation-advice-on-priority-groups-for-covid-19-vaccination-30-december-2020 (accessed March 12, 2021).
